# Oxygen Evolution Reaction at Microporous Pt Layers: Differentiated Electrochemical Activity between Acidic and Basic Media

**DOI:** 10.1038/s41598-017-15688-9

**Published:** 2017-11-13

**Authors:** Taejung Lim, Moonchang Sung, Jongwon Kim

**Affiliations:** Department of Chemistry, Chungbuk National University, Cheongju, Chungbuk 28644 South Korea

## Abstract

Nanoporous electrodes have received great attention because of their unique electrochemical properties. Here, the electrocatalytic oxygen evolution reaction (OER) activities at porous Pt layers with pore dimensions in the microporous range were examined. The OER activity of the porous Pt layers in acidic media increased as the porosity of the Pt layers increased, and the highest OER activity possessed an overpotential that was 270 mV lower than that of a bulk flat electrode. The porous Pt layers did not exhibit electrocatalytic enhancement for OER in basic media, wherein the surface area of the pores was not utilized for OER. The differentiated OER activity of the porous Pt layers demonstrated the different accessibility of reactants in OER: water and hydrated hydroxide ions. The roles of the pores in the Pt layers during OER were investigated using different Pt structures. The work will give insight into the electrochemistry of microporous electrode structures.

## Introduction

Nanoporous electrodes have received great attention because of their unique electrochemical properties and applications in electrocatalysis and electroanalysis^1,2^. Extensive research has been devoted to the preparation of nanoporous electrode materials. Nanoporous Au electrodes have been prepared by dealloying Au-Ag or anodizing Au electrodes^3,4^, and their electrochemical applications have been reported^5^. Nanoporous Pt electrodes have been prepared by the electrodeposition of Pt using self-assembled surfactants and polystyrene spheres as templates^6^. Unique electrocatalytic activities have been demonstrated by nanoporous Au and Pt electrodes for electrochemical reactions, such as oxygen reduction and methanol oxidation^7^. Nanoporous electrodes that have been reported thus far have pore dimensions greater than several nanometers; thus, these electrodes possess ‘mesopores’^8^. The mesopores present in these electrodes are large enough for electrochemically active species to diffuse into the pores for electrochemical reactions. In contrast, nanoporous electrodes with pore dimensions less than 2 nm (so called ‘micropores’) have seldom been investigated for electrochemical reactions because of their small pore sizes. An anomalous electrochemical capacitance was observed for microporous carbon with pore sizes less than 1 nm^9^.

The oxygen evolution reaction (OER) is an important electrochemical reaction limiting the efficiency of electrochemical water splitting, which provides an attractive route for the production of sustainable energy sources^10^. Numerous efforts have been devoted to the development of efficient OER electrocatalysts^11^. Most previous research has focused on the optimization of the composition of metal electrocatalysts for OER, whereas the effect of the electrode structure on OER activity has been less frequently investigated. Reier and coworkers reported a comparative study between nanoparticles and bulk electrodes for electrocatalytic OER^12^. Recently, Gewirth and coworkers showed that Ni-based porous electrodes prepared by electrodeposition exhibited highly active and stable electrocatalytic activities for OER^13^. In the present work, we investigated the electrocatalytic OER activity at electrochemically deposited Pt layers. We previously reported that flat Pt layers retaining an unusually large electrochemical surface area (ESA) can be prepared by electrodeposition^14^. TEM analysis revealed that the large ESA was ascribed to the channel-like pores present inside the Pt layers that are smaller than several nanometers. It was also shown that the dimensions of the pores were too small to utilize the large ESA for electrochemical reactions, such as glucose oxidation. In other words, the electroactive species could not diffuse into the pores for electron transfer at the pore surfaces; thus, the porous Pt can be regarded as a microporous system. Interestingly, we observed that the electrodeposited Pt layers exhibited significantly enhanced electrocatalytic activity for OER compared to a bulk Pt electrode. The OER activity increased as the porosity of the Pt layers increased, which signifies that the enlarged ESA can be utilized for OER. The influence of the porous structure on OER activity on the porous Pt layers was examined, and the mechanistic details of OER in the pores were discussed.

## Results and Discussion

**Figure 1:**
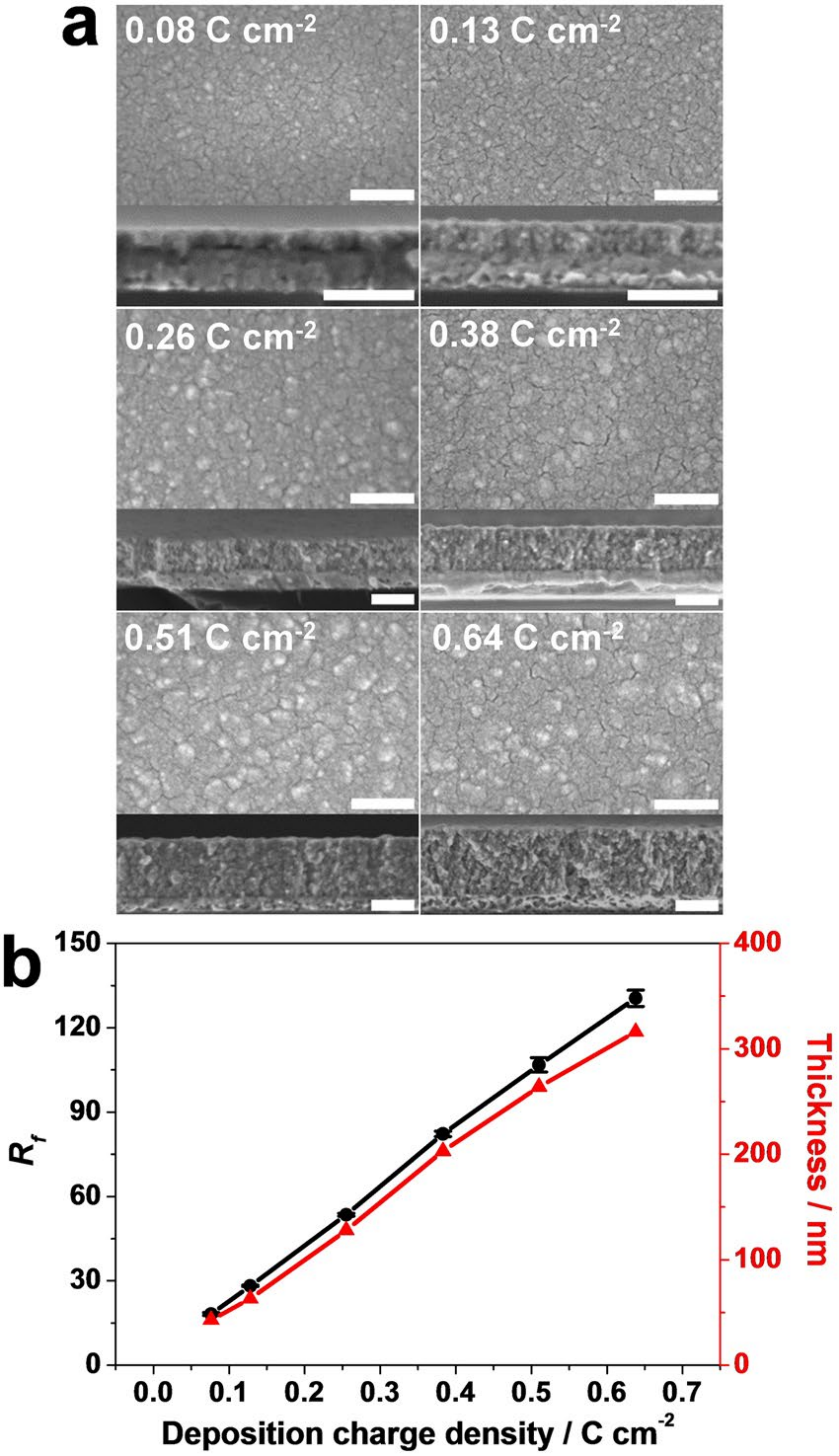
(**a**) Top and cross-sectional SEM images of the Pt layers electrodeposited at 0.2 V (vs. Ag/AgCl) in solutions containing 30 mM K_2_PtCl_4_ and 0.1 M H_2_SO_4_ with various deposition charge densities. Scale bar = 200 nm. (**b**) Dependence of the *R*_*f*_ value and the thickness of the Pt layers on the deposition charge density. Each 5 data of the *R*_*f*_ were used for error bar.

Figure 1a shows SEM images of the Pt layers electrodeposited with different charge densities. The top SEM images reveal that the surface morphologies exhibit flat features with some bumps and cracks regardless of the deposition charge density. As the charge density increases, the thickness of the Pt layers linearly increases from 40 nm to 320 nm, as shown in Fig. 1b. Despite the flat structures of the electrodeposited Pt layers, the ESA of the Pt layers was determined to be very large. The ESA of Pt layers were measured from cyclic voltammograms obtained in 0.1 M H_2_SO_4_ (Supplementary Information, Figure S1), from which the ESA of Pt layers was calculated by integrating the charge consumed for the hydrogen monolayer adsorption on Pt surfaces (210 mC cm^−2^ refer to the Supplementary Information for the detailed ESA evaluation)^15^. Roughness factors (*R*_*f*_ values, ESA divided by the geometric area) plotted as a function of the deposition charge density are shown in Fig. 1b. The *R*_*f*_ value linearly increases with the deposition charge density ranging from 19 to 130. We previously reported that high roughness factors obtained on flat Pt layers originated from channel-like small pores present inside the Pt layers as evidenced from the TEM analysis of the electrodeposited Pt layers^14^. These pores are not accessible to redox-active species, such as glucose and Fe(CN)_6_^3−^; thus, the enlarged surfaces of the Pt layers do not participate in the electrochemical reactions. We speculate that the pores inside the Pt layers might participate in OER with the water solvent reacting as reagent molecules.

**Figure 2:**
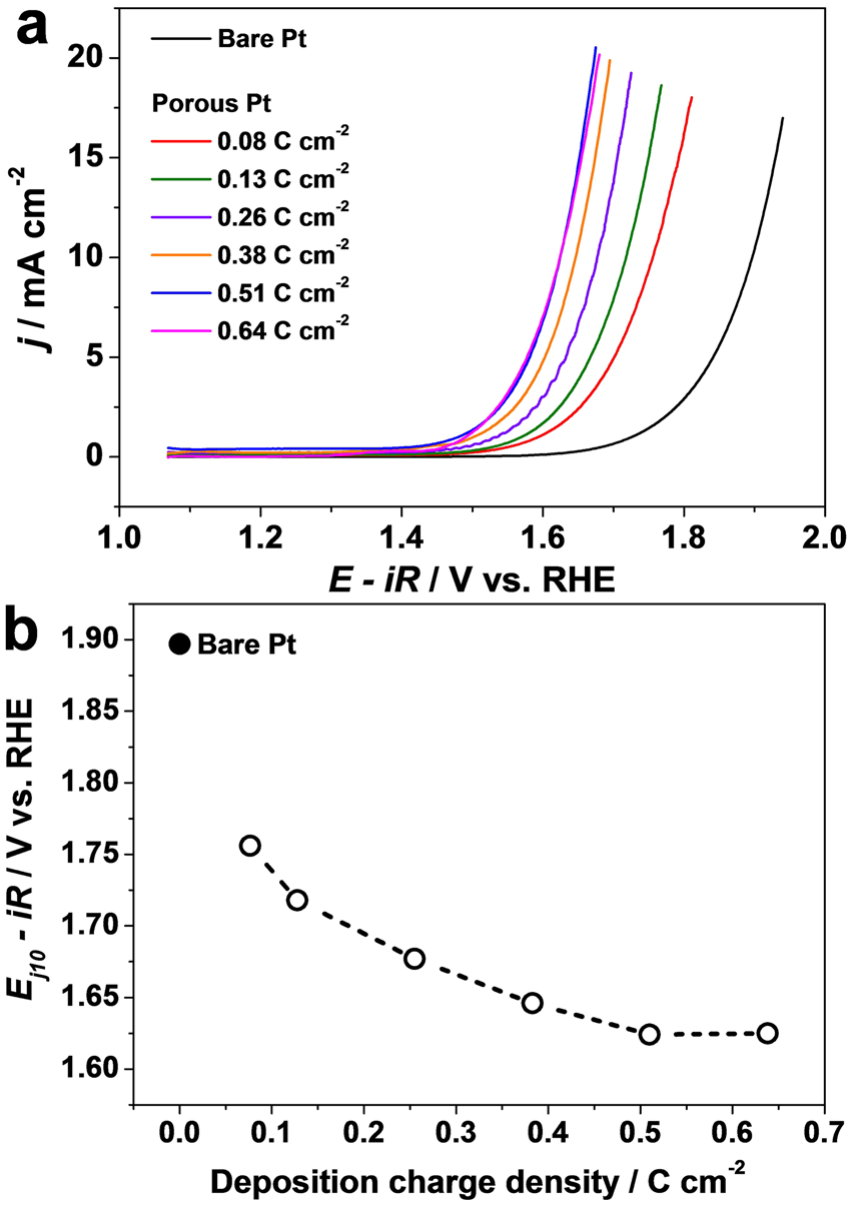
(**a**) OER polarization curves (*iR* corrected) of a bare Pt electrode and the porous Pt electrodes in 0.1 M HClO_4_ recorded with 6 mV s^−1^ and 1600 rpm. (**b**) OER potentials at the porous Pt surfaces with 10 mA cm^−2^.

Figure 2a shows the OER polarization curves (*iR* corrected) obtained on the porous Pt layers electrodeposited with different deposition charge densities. The electrochemically deposited Pt layers exhibit significantly enhanced OER activities compared to that of a flat bulk Pt electrode. As the electrodeposition charge density increases, the OER activity of the porous Pt layers gradually increases. The potentials corresponding to an OER current density of 10 mA cm^−2^ (denoted as E_j10_) were plotted as a function of the deposition charge density (Fig. 2b). The E_j10_ value of the porous Pt layers electrodeposited with 0.08 C cm^−2^ is 1.76 V, corresponding to a 140 mV lower overpotential for OER than that of a bare Pt electrode. The OER overpotential on the Pt layers further decreases as the deposition charge density increases, and the E_j10_ value of the porous Pt layers electrodeposited with 0.51 C cm^−2^ reaches 1.62 V (270 mV lower overpotential than that of a bare Pt electrode). No further decrease in overpotential was observed at higher deposition charge densities. The enhanced OER activity observed on the electrodeposited, porous Pt layers is quite significant and is better than that of a bulk Pd or Rh electrode^16^.

The enhanced OER activities obtained on the porous Pt layers with high roughness factors imply that the increased ESA of the Pt layers effectively contributes to the OER activity. Since no notable morphological features are present on the surfaces, the porous structures in the Pt layers might be responsible for the enhancement in the OER activity. Nanoporous electrodes exhibit unique electrochemical behaviors compared to the corresponding flat electrodes due to their distinct structural features, such as the nanoconfinement effect^1,2^. In nanoporous electrodes, interactions between the electroactive species and electrode surfaces are more frequent, which results in enhanced electrocatalytic performance. However, the unique electrochemical behavior in the nanopores requires sufficient pore dimensions for redox species to diffuse into the pores. The pore dimensions inside the electrodeposited Pt layers are too small to be accessible to redox species; thus, no electrochemical enhancements should be achieved. Interestingly, a significant enhancement in the electrochemical activity for OER was observed on the porous Pt layers. Moreover, the degree of activity enhancement increased with the porosity (*R*_*f*_) of the Pt layers. We examined the electrochemical response of the porous Pt layers for the oxygen reduction reaction (ORR) (Supplementary Information, Figure S2); however, the ORR activity was not enhanced with the porosity Pt layers. These results signify that the pores inside the Pt layers are effectively utilized in electrochemical OER with water molecules participating as reactants.

To examine the electrode kinetics of OER, Tafel plots of a bare Pt electrode and the Pt layers (Supplementary Information, Figure S3) were obtained from Fig. 2a. The Tafel slope of the bare Pt electrode was 162 mV dec^−1^, which is similar to the slopes reported in previous works^16^. The Tafel slope of the porous Pt layers electrodeposited with 0.08 C cm^−2^ was 153 mV dec^−1^, and smaller slopes were obtained at the Pt layers with increased deposition charge densities. The electrode kinetics at the Pt layers are enhanced compared to a bare Pt electrode; however, the enhancement is not significant.

**Figure 3:**
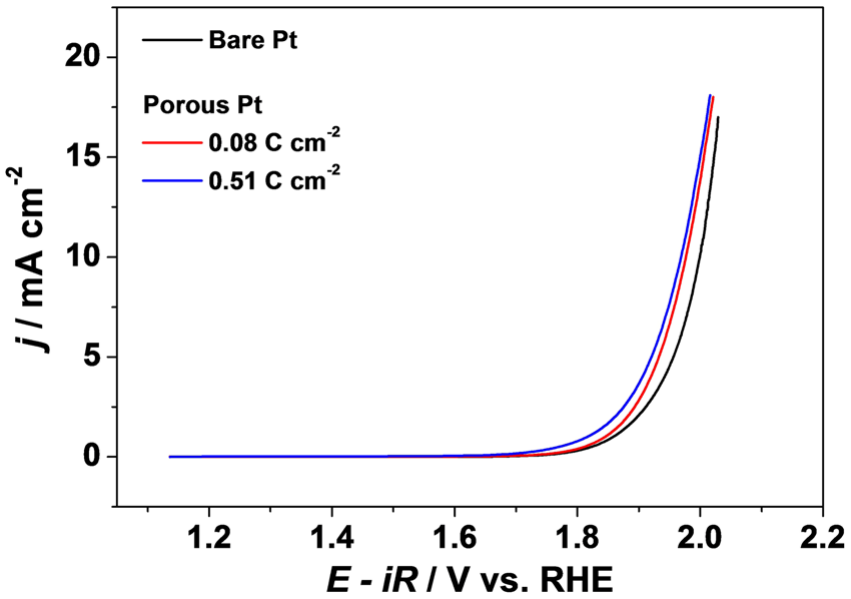
OER polarization curves (*iR* corrected) of a bare Pt electrode and the porous Pt electrodes in 1 M KOH recorded with 6 mV s^−1^ and 1600 rpm.

Figure 3 shows the OER polarization curves obtained in 1 M KOH solutions on the porous Pt layers and a bulk Pt electrode. In contrast to the results obtained in acidic media (0.1 M HClO_4_), the OER activities of the porous Pt layers are not significantly enhanced compared to that of a bulk Pt electrode. More interestingly, no difference was observed in the OER activities between the porous Pt layers electrodeposited with deposition charge densities of 0.08 C cm^−2^ and 0.51 C cm^−2^. These results indicate that the pores inside the Pt layers are not utilized in electrochemical OER in basic media, in contrast to acidic media. In acidic media, OER proceeds as:

1 $$2{{\rm{H}}}_{2}{\rm{O}}\to {{\rm{O}}}_{2}+4{{\rm{H}}}^{+}+4{{\rm{e}}}^{-}$$

where water molecules function as reactants at the surfaces. On the other hand, OH^−^ ions serve as reactants in basic media, as shown below.

2 $$4{{\rm{OH}}}^{-}\to 2{{\rm{H}}}_{2}{\rm{O}}+4{{\rm{e}}}^{-}+{{\rm{O}}}_{2}$$

Since the OH^−^ ions are solvated by water molecules, the hydrated radii of the OH^−^ ions are much greater than water molecules^17^. The different OER activity on the porous Pt layers between acidic and basic media indicates that the pores inside Pt layers are large enough to allow access to water molecules but too small to allow access to hydrated OH^−^ ions. The ESA (*R*_*f*_) of the Pt layers did not change after the OER measurements in basic media, confirming that the porosity of the Pt layers is conserved (Supplementary Information, Figure S4). The OER activity observed on the porous Pt layers was recovered in acidic media after the OER measurements in basic media. The OER activities between the porous Pt layers electrodeposited with 0.08 C cm^−2^ and 0.51 C cm^−2^ and a bulk Pt electrode are significantly different in acidic media (Supplementary Information, Figure S5). The differentiated activity between acidic and basic media indicates that the pores inside the Pt layers play a role in the enhancement of electrochemical OER.

**Figure 4:**
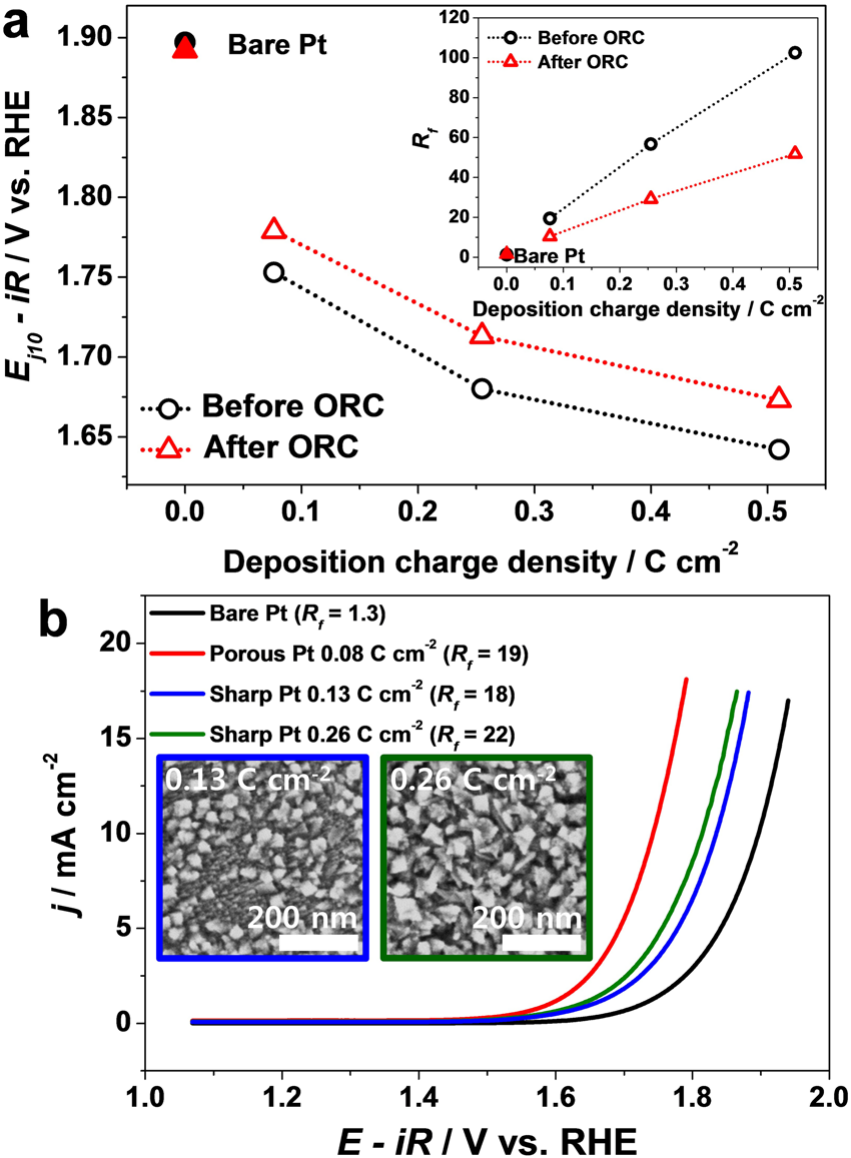
(**a**) OER potentials at 10 mA cm^−2^ of the porous Pt before and after ORCs recorded with 6 mV s^−1^ and 1600 rpm in 0.1 M HClO_4_. The Inset shows *R*_*f*_ values of a bare Pt electrode and the porous Pt before and after the ORCs. (**b**) OER polarization curves (*iR* corrected) of a bare Pt electrode and the sharp Pt structures in 0.1 M HClO_4_ recorded with 6 mV s^−1^ and 1600 rpm. Insets show the SEM images of sharp Pt structures.

To further examine the effect of the pores present in the Pt layers on the OER activity, we reduced the *R*_f_ value of the porous Pt layers by electrochemical treatment. The as-prepared porous Pt layers were subjected to electrochemical oxidation-reduction cycles (ORCs) in 0.1 M H_2_SO_4_, which resulted in a decrease of the ESA of the porous Pt layers (Supplementary Information, Figure S6). The formation and dissolution of the oxides on the Pt surface induced coalescence of the pores inside the Pt layers, resulting in a decrease of the ESA of the Pt layers. The *R*_*f*_ values of the porous Pt layers before and after the ORCs are compared in the inset of Fig. 4a, wherein the *R*_*f*_ value of the porous Pt layers decreased 50% after the ORCs regardless of the deposition charge density. For a bare Pt electrode, the ESA slightly increased due to a roughening of the Pt surface during the ORCs^18^.

The OER polarization curves of the porous Pt layers obtained after the ORCs show that the OER activity decreased compared to the as-prepared Pt layers (Supplementary Information, Figure S7). Figure 4a compares the E_j10_ values during OER measured on the porous Pt layers before and after the ORCs. The flat Pt electrode did not exhibit a change in OER activity after the ORCs. The E_j10_ value of the porous Pt layers electrodeposited with 0.08 C cm^−2^ increased by 26 mV after the ORCs. The porous Pt layers electrodeposited with greater charge densities exhibited slightly greater increases in OER overpotentials. Tafel plots of the porous Pt layers were obtained after the ORCs (Supplementary Information, insets in Figure S7), showing slightly increased Tafel slopes compared to those obtained before the ORCs. However, the bare Pt electrode exhibited no change in Tafel slope. These results show that reducing the *R*_*f*_ value (decrease in ESA) of the porous Pt layers results in a decrease in the OER activity, which supports that the pores present in the Pt layers contribute to the OER activity.

Although the OER activity decreases as the porosity of the Pt layers reduces in acidic media, the degree of activity decrease is small considering that the *R*_*f*_ value of the Pt layers was reduced by 50%. To elucidate the origin of the enhancement of the OER activity at the porous Pt layers, we examined the OER activity of electrodeposited Pt structures with sharp features retaining similar *R*_*f*_ value as the porous Pt layers. Pt nanostructures with sharp edges were electrodeposited at −0.24 V (vs. Ag/AgCl) from solutions containing 30 mM K_2_PtCl_4_ and 0.1 M H_2_SO_4_, and the SEM images of these nanostructures are shown in the inset of Fig. 4b. The *R*_*f*_ value of the sharp Pt nanostructure electrodeposited with 0.13 C cm^−2^ was 18 (Supplementary Information, Figure S8), which is similar to that of the porous Pt layers electrodeposited with 0.08 C cm^−2^. The OER activity of the sharp Pt nanostructure increased compared to a flat bulk Pt electrode; however, this activity was much lower that the OER activity of the porous Pt layers with a similar *R*_*f*_ value (Fig. 4b). The *R*_*f*_ value of the sharp Pt nanostructure electrodeposited with 0.26 C cm^−2^ was 22, but the OER activity slightly increased. These results indicate that the enhancement of the OER activity of the porous Pt layers compared to that of a flat bulk Pt electrode does not originate solely from the increase in ESA (*R*_*f*_); however, the porous nature of the Pt layers significantly contributes to the OER activity. Fresh Pt surfaces prepared by electrodeposition of Pt or nanoscale features on the porous Pt layers results in an enhancement of the OER activity compared to a mechanically polished bulk Pt electrode.

## Conclusions

We investigated the electrocatalytic OER activity at electrochemically deposited porous Pt layers. The micropores present inside the Pt layers were effectively utilized for OER in acidic media, which resulted in increased OER activity compared to that of a bulk Pt electrode. The OER activity increased when the porosity of the Pt layers increased by applying a higher charge density during the electrodeposition of the Pt layers. In basic media, however, the porous Pt layers did not exhibit electrocatalytic enhancement for OER, indicating that the micropores do not participate in OER. The differentiated electrochemical activity of the porous Pt layers between acidic and basic media indicates that the micropores inside the Pt layers are accessible to reactants (water molecules) in acidic media but not to hydrated OH^−^ ions in basic media. The roles of the pores in the Pt layers toward OER were further confirmed by control experiments using Pt layers with reduced pores and sharp Pt nanostructures with similar roughness as the porous Pt layers. The results shown in this work will give insights into the electrochemistry at porous electrode structures.

## Methods

### Preparation of Pt electrodes

All solutions were prepared using purified water (Milli-Q, 18.2 MΩ cm^−1^). Pt rotating disk electrodes with 2 mm disk diameter (Pine Research Instruments) and Pt foils (0.02-in thick, Alfa Aesar) were used as the working electrode for OER measurements and scanning electron microscopy (SEM) observations, respectively. The Pt electrodes were polished with a 1.0-μm alumina suspension and then with a 0.05-μm suspension on Microcloth (Buehler) to obtain a mirror finish. All electrochemical depositions and measurements were performed using a CHI 750E (CH Instruments). Pt-mesh counter electrodes and Ag/AgCl (3 M NaCl) reference electrodes were used. Electrodeposition of the Pt layers was performed under a constant potential of 0.2 V (vs. Ag/AgCl) in solutions containing 30 mM K_2_PtCl_4_ and 0.1 M H_2_SO_4_. The sharp Pt structures were electrodeposited at −0.24 V (vs. Ag/AgCl) in the same solution used for electrodeposition of the Pt layers. The electrolyte solutions were purged with N_2_ before the electrochemical depositions.

### Physical and electrochemical characterizations

SEM images were obtained using an ULTRA PLUS field-emission scanning electron microscope (Carl Zeiss). All potentials were converted to the potential scale of the reversible hydrogen electrode (RHE) unless otherwise specified. The ESA of Pt layers were calculated by integrating the charge consumed for the hydrogen monolayer adsorption from cyclic voltammograms obtained in 0.1 M H_2_SO_4_. The OER and ORR polarization curves were recorded in N_2_-saturated 0.1 M KClO_4_, 0.1 M HClO_4_ (Merck), and 1.0 M KOH (Sigma Aldrich) at rotating rates of 1600 rpm, using an AFMSRCE electrode rotator (Pine Research Instrument). The OER measurements were conducted with *iR* compensation. Tafel plots and Tafel slopes were derived from OER polarization curves with the linear portions fitted to Tafel equation (η = b log *j* + a, where η is overpotential, *j* is the current density, and b is Tafel slope).

## Electronic supplementary material

Supplementary Information
