# Supplementary material for: Oxygen Evolution Reaction at Microporous Pt Layers: Differentiated Electrochemical Activity between Acidic and Basic Media
Source: Sci Rep. 2017 Nov 13;7:15382. doi: 10.1038/s41598-017-15688-9 (PMC5684209; doi:10.1038/s41598-017-15688-9)
Supplement: Supplementary file 1 — Supplementary Information [file 41598_2017_15688_MOESM1_ESM.pdf]

Supplementary Information

Oxygen Evolution Reaction at Microporous Pt Layers: Differentiated Electrochemical Activity between Acidic and Basic Media

Taejung Lim, Moonchang Sung, and Jongwon Kim*

*Department of Chemistry, Chungbuk National University, Cheongju, Chungbuk, 28644 (South Korea)

E-mail: JongwonKim@chungbuk.ac.kr

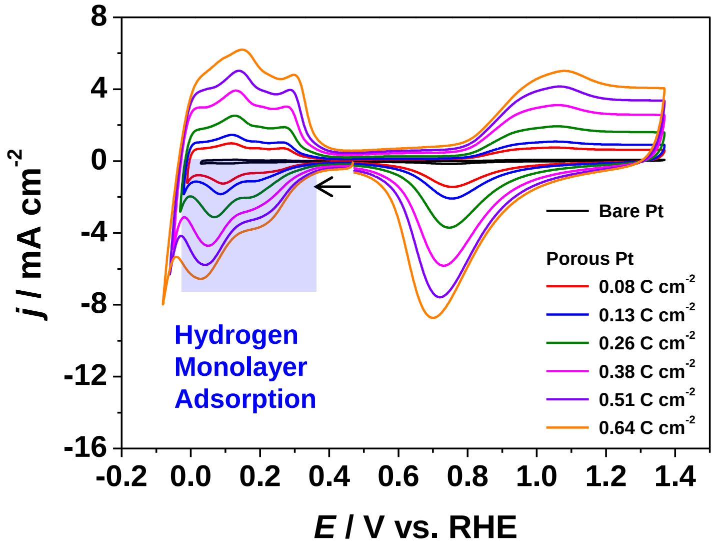

Figure S1. Cyclic voltammograms of the porous Pt layers electrodeposited with various charge densities obtained in 0.1 M H_2SO_4. Scan rate = 50 mV s^{-1}.

The ESA of Pt layers were measured from cyclic voltammograms obtained in 0.1 M H_2SO_4 (Figure S1), from which the ESA of Pt layers was calculated by integrating the charge consumed for the hydrogen monolayer adsorption on Pt surfaces using following equations.

$$Q_H = \int_{t_i}^{t_f} I\,dt - Q_{dl} \qquad A^* = \frac{Q_H}{Q_H^*}$$

Q_H = the charge associated to the hydrogen monolayer adsorption on Pt

Q_H* = the charge associated to the hydrogen monolayer adsorption on Poly Pt, based on density of atoms = 210 μC cm^{-2}

Q_{dl} = the charge associated to double layer capacitive processes in the hydrogen adsorption region

A* = electrochemical surface area (ESA)

A = geometric surface area of RDE substrate = 0.196 cm^2

Table S1. Electrochemical surface area(ESA) and R_f values of bare Pt and the porous Pt electrodes calculated with Figure S1.

Deposition Charge (C cm^{-2})	A* (cm^2)	R_f (A*/A)
Bare Pt	0.250	1.28
0.08	3.73	19.05
0.13	5.63	28.70
0.26	10.4	52.93
0.38	16.57	74.55
0.51	21.57	110.06
0.64	26.10	133.14

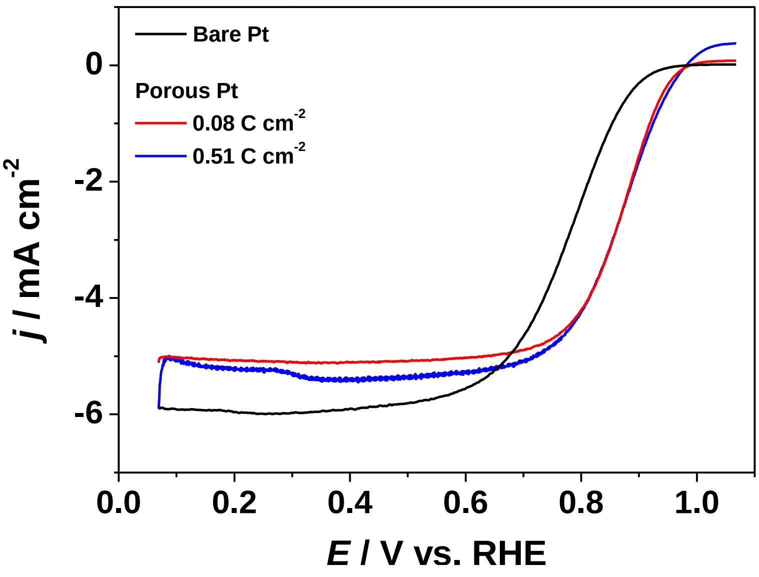

Figure S2. ORR polarization curves of a bare Pt electrode and the porous Pt electrodes in 0.1 M $HClO_4$ recorded with 6 mV s^{-1} and 1600 rpm.

Figure. S2 shows that the enhanced ORR activity of the porous Pt layers compared to the bare Pt electrode does not originate from the pore effect but from the fresh Pt surface prepared by electrodeposition of Pt or from nanoscale features on the porous Pt layers (refer to the conclusion of manuscript).

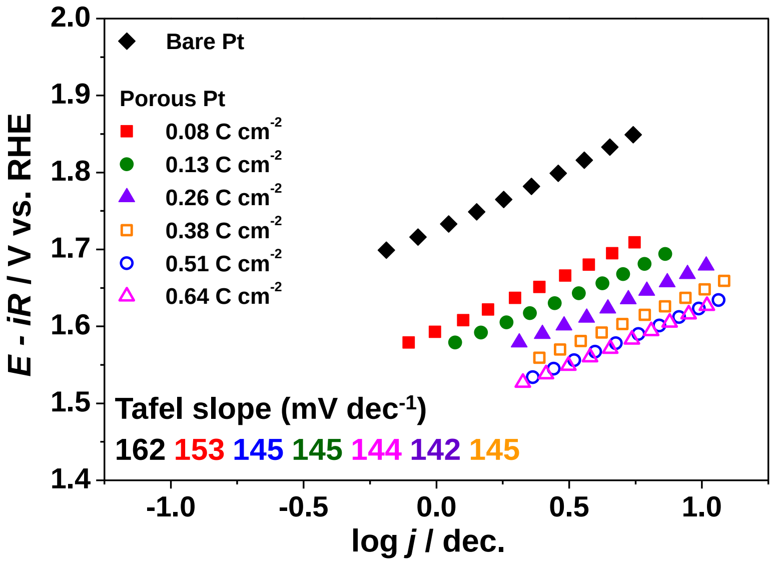

Figure S3. Tafel plots of the porous Pt layers electrodeposited with different charge densities obtained from the *iR* corrected OER polarization curves in 0.1 M $HClO_4$.

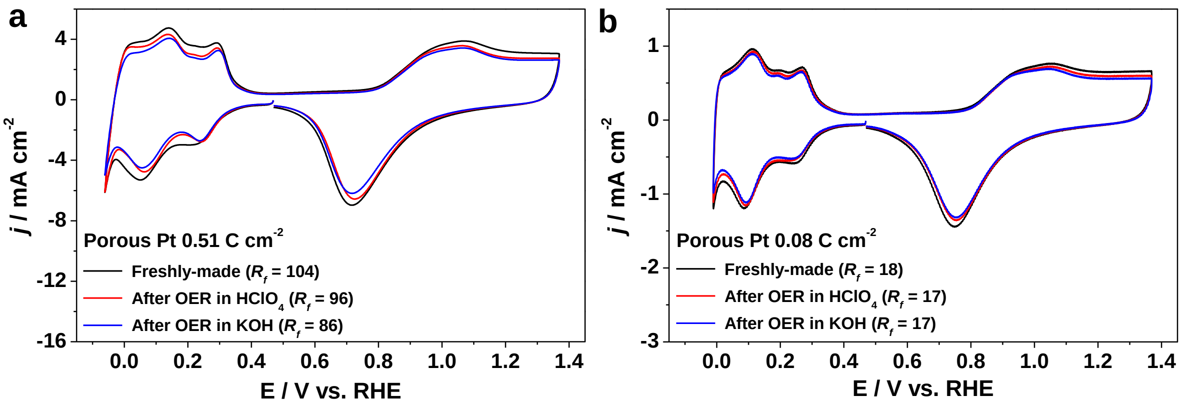

Figure S4. Cyclic voltammograms of the porous Pt layers in 0.1 M H_2SO_4 before and after OER measurements in basic media. Scan rate = 50 mV s^{-1}.

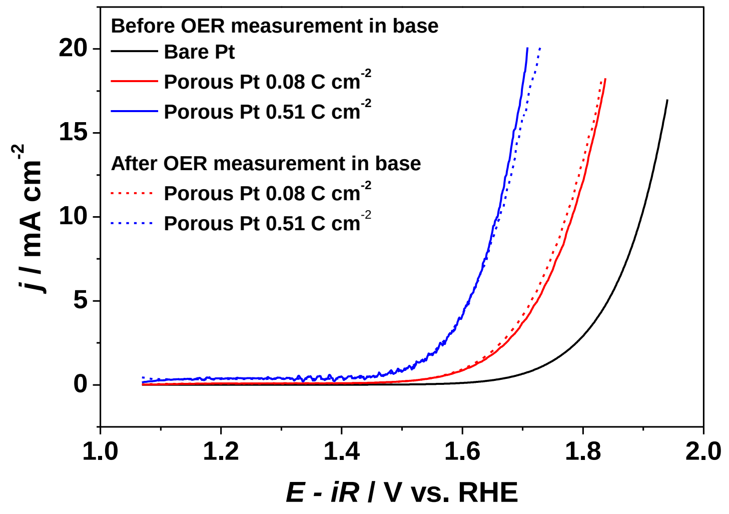

Figure S5. OER polarization curves (*iR* corrected) of the porous Pt layers in 0.1 M $HClO_4$ before and after OER measurements in basic media recorded with 6 mV s^{-1} and 1600 rpm.

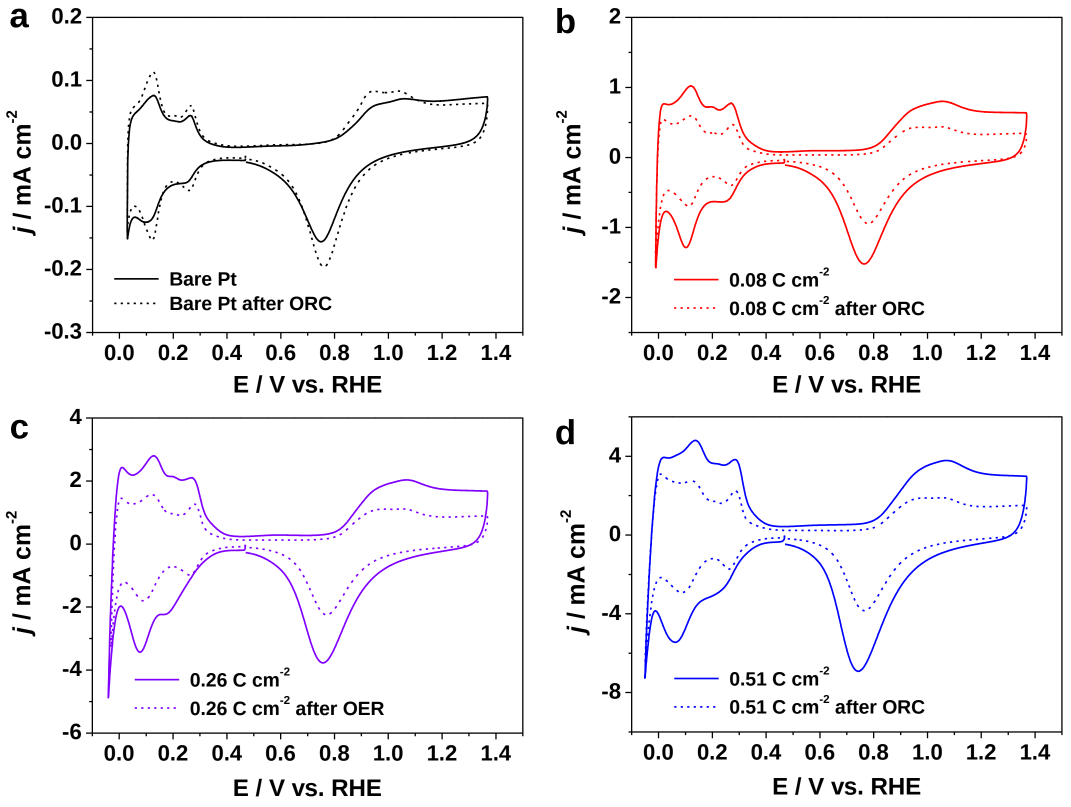

Figure S6. Cyclic voltammograms of the porous Pt layers in 0.1 M H_2SO_4 before and after the ORCs: (a) bare Pt, (b) 0.08 C cm^{-2}, (c) 0.26 C cm^{-2}, and (d) 0.51 C cm^{-2}. Scan rate = 50 mV s^{-1}.

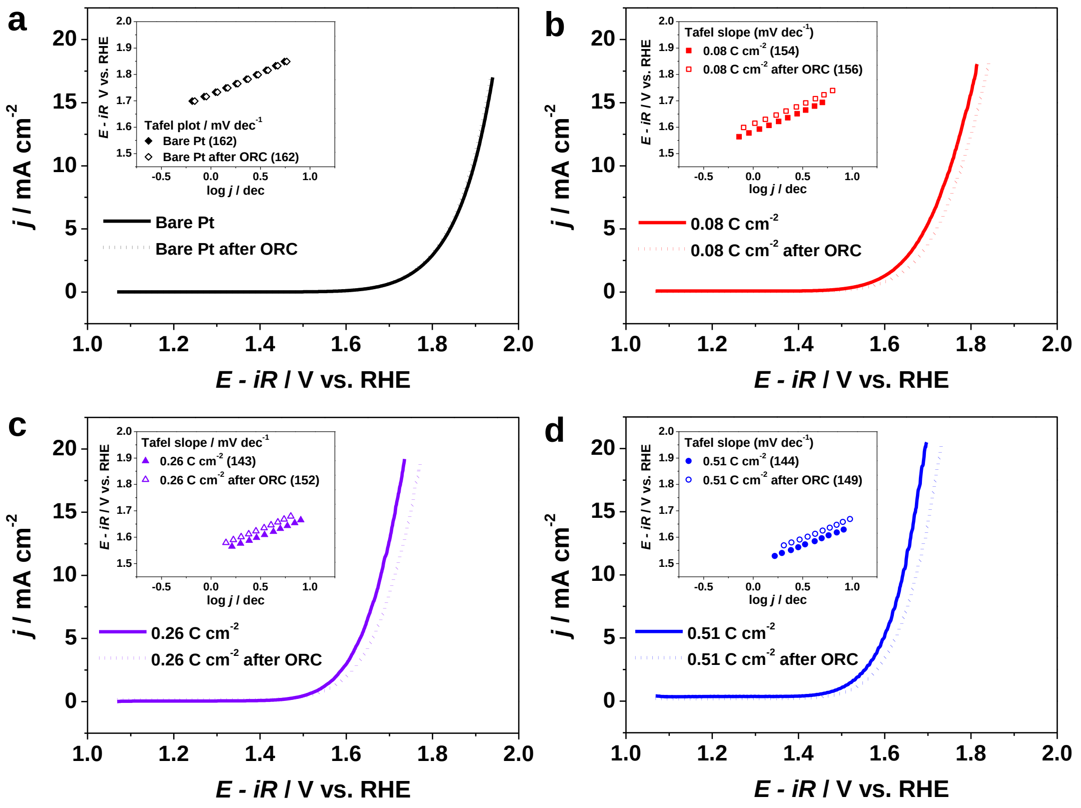

Figure S7. OER polarization curves (*iR* corrected) of a bare Pt electrode and the porous Pt layers in 0.1 M $HClO_4$ before and after the ORCs recorded with 6 mV s^{-1} and 1600 rpm (Inset: Tafel plots before and after the ORCs): (a) bare Pt, (b) 0.08 C cm^{-2}, (c) 0.26 C cm^{-2}, and (d) 0.51 C cm^{-2}.

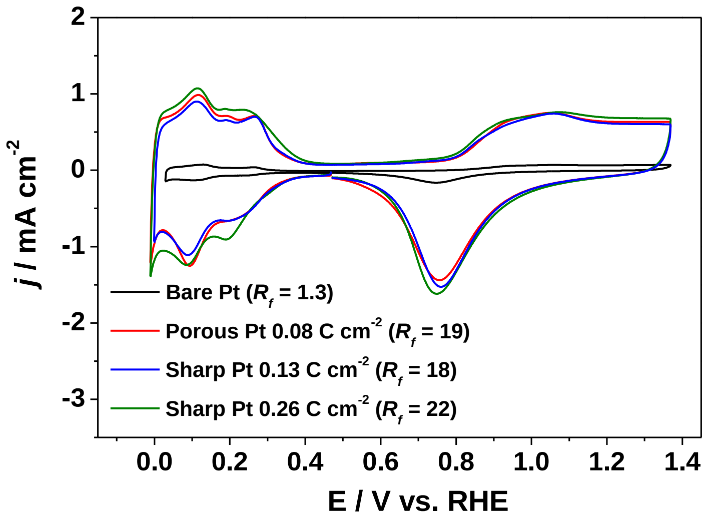

Figure S8. Cyclic voltammograms of the Pt electrodes in 0.1 M H_2SO_4. Scan rate = 50 mV s^{-1}.
